# Factors Associated with the Leisure-Time Physical Activity (LTPA) during the First Trimester of the Pregnancy: The Cross-Sectional Study among Pregnant Women in Serbia

**DOI:** 10.3390/ijerph17041366

**Published:** 2020-02-20

**Authors:** Jovana Todorovic, Zorica Terzic-Supic, Vesna Bjegovic-Mikanovic, Pavle Piperac, Stefan Dugalic, Miroslava Gojnic-Dugalic

**Affiliations:** 1 Institute of Social Medicine, Faculty of Medicine, University of Belgrade, Dr Subotica 15, 11000 Belgrade, Serbia; zorica.terzic-supic@med.bg.ac.rs (Z.T.-S.); vesna.bjegovic-mikanovic@med.bg.ac.rs (V.B.-M.); 2 Department of Humanities, Faculty of Medicine, University of Belgrade, Pasterova 2, 11000 Belgrade, Serbia; pavle.piperac@med.bg.ac.rs; 3 Clinic of Obsterics and Gynecology, Clinical Centre of Serbia, Belgrade, 11000 Beograd, Serbia; stef.dugalic@gmail.com (S.D.); miroslavagojnicdugalic@yahoo.com (M.G.-D.)

**Keywords:** physical activity, pregnancy, factors, walking

## Abstract

*Background*: The benefits of physical activity during pregnancy include lower maternal weight gain, a lower likelihood of gestational diabetes, low back pain, preeclampsia, preterm delivery, caesarian delivery, and macrosomia. This study aimed to examine the factors associated with insufficient leisure-time physical activity (LTPA) during the first trimester. *Methods*: A cross-sectional study was conducted at the Clinic for Obstetrics and Gynecology of Clinical Center of Serbia, Belgrade, between January and June of 2018. The final analyses included 162/175 pregnant women. The questionnaire was used to obtain social characteristics, pregnancy, and lifestyle characteristics (Pregnancy Risk Assessment Monitoring System—PRAMS), pre-pregnancy LTPA (International Physical Activity Questionnaire—IPAQ), and LTPA during the first trimester (Pregnancy Physical Activity Questionnaire—PPAQ). Women were classified into two groups of sufficient and insufficient LTPA during the first trimester based on the recommendations of the World Health Organization. Multivariate logistic regression analysis was applied. *Results*: A total of 27.2% of the women had insufficient LTPA during pregnancy. Insufficient LTPA during pregnancy was associated with <12 years of education (OR: 2.3, 95% CI: 1.05–5.04), self-rated financial status as poor (OR: 0.34, 95% CI: 0.14–0.79), and hours spent walking before pregnancy (OR: 0.87, 95% CI: 0.77–0.99). *Conclusions*: Our results can help direct health care professionals advice for women who are planning pregnancy towards walking as it seems to be sustained during pregnancy.

## 1. Introduction 

Physical activity (PA) provides health benefits in all stages of life, it can improve quality of life, mood, and general health, manage obesity and obesity-related problems [1,2,3,4,5]. Traditionally, the widespread opinion was that pregnant women should avoid every type of PA, as it was thought that PA reduces placental circulation and therefore, increases the risk for preterm delivery, growth retardation, and miscarriages [6], although The American College of Obstetricians and Gynecologists (ACOG) gave the recommendation for PA during pregnancy back in 1994 [7]. The benefits of PA during pregnancy were confirmed in recent research as well [8,9,10]. 

The recommendations of ACOG were reaffirmed in 2009 and 2015, stating that all pregnant women without contraindications (medical or obstetric) for PA should engage in 30 min of moderate-intensity PA per day on most or all days a week [11]. Similarly, the Society of Gynecologists and Obstetricians of Canada and the Canadian Society for Exercise physiology gave recommendations for a minimum of 150 min of moderate-intensity PA per week [12]. The World Health Organization (WHO) gave recommendations in 2011 for minimal energy expenditure of 600 MET (metabolic equivalents of task)/min/week for health benefits for all adults, including pregnant women [13]. 

Studies on the topic of PA during pregnancy showed that it could reduce maternal weight gain and the risk for development of gestational diabetes mellitus (GDM) [14,15,16,17,18]. The studies highlight the importance of early initiation of PA during pregnancy [12]. The initiation of the PA during the first trimester is associated with lower risk for development of gestational diabetes [15,16,17,18]. Additionally, early initiation of PA during pregnancy is associated with a lower likelihood of preeclampsia [19,20] and better sleep quality [21]. This leads to further need for examination of factors that influence the PA level during the first trimester.

Other health benefits of PA during pregnancy include relief of the symptoms of low back pain, which is very common and affects around 60% of pregnant women [22,23,24], a decrease in the likelihood of caesarian delivery and postpartum recovery time [11,25,26,27]. Studies have also shown that physically active pregnant women have a lower chance for the development of depression and have lower anxiety levels [28,29,30,31,32]. The studies on the influence of the in utero environment show that it has effects on infants and throughout childhood into adulthood [33], which is in accordance with developmental origins hypothesis. PA during pregnancy has a positive effect on the offspring which was shown in the Danish National Birth Cohort study, with lower body mass index (BMI) and lower frequency of obesity at the age of 7 [34]. The exposure to exercise during pregnancy is associated with cardiac and neuromotor development of the infants, as studies have shown lower heart rate and increased heart rate variability among infants, which is the same as among adults who are exercised trained [35]. Further, children with slower heart rates are shown to have higher psychomotor and language developmental outcomes at one, two, and three years of age [33]. 

Most women do not meet pregnancy PA recommendations, but it seems that women who are physically active before pregnancy have a higher likelihood of remaining active during pregnancy [28]. Among factors associated so far with the reduction of PA are lower socioeconomic status and a greater number of children [36]. Additionally, perceived barriers to PA among pregnant women were symptoms of pregnancy as women often state that tiredness and nausea were the factors that hindered PA along with family and household obligations or fear of injuries [37]. Another commonly reported reason for the reduction of PA during pregnancy is discomfort during exercise. This discomfort usually comes during the second trimester, when there are changes in posture, the shift of point of gravity, and progressive lordosis [11]. 

The majority of studies which examined the influence of PA interventions among pregnant women included participants in the second trimester, or late in the first trimester [38]. Consequently, the factors associated with PA levels in the second trimester are well understood [39]. However, PA in the first trimester is associated with some benefits such as positive effects on prevention of GDM, preeclampsia, and sleep disturbances. It is important to examine which factors are associated with PA levels during the early pregnancy. 

The aim of this study was to explore the factors associated with insufficient PA during the first trimester.

## 2. Methods

This was a cross-sectional study conducted at the Clinic for Obstetrics and Gynecology of Clinical Center of Serbia, Belgrade, between January and June of 2018. 

### 2.1. Population

**Figure 1:**
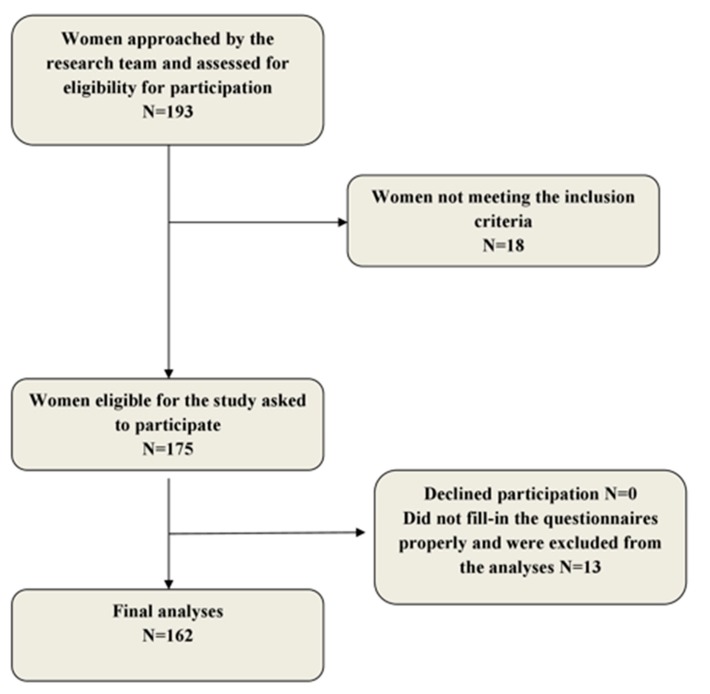
Flow-chart of recruitment and participation.

The study included 175 pregnant women who had a regular appointment with the gynecologist, at the end of their first trimester (12th week of gestation). All women with a singleton pregnancy and without existing obstetric and medical contraindications for PA during pregnancy were included in the study. Exclusion criteria were: existing medical or obstetric contraindication for PA during pregnancy [40], heavy smoking, and mental inability to understand and fill in the questionnaire. Eligibility of women was assessed by the members of the research team (lead researcher and on-call gynecologist) Once eligibility was confirmed, potential participants were invited to participate in the study. Participants were asked to fill-in the hard copy of questionnaires. Participants could ask the lead researcher if they had any questions regarding the completion of the questionnaire. The details on the recruitment of the participants are presented in Figure 1.

### 2.2. Ethical Approval 

All participants were given an oral and written description of the study, its processes and aims and all of them gave written consent for participation. The ethical committee of the Belgrade Medical Faculty gave consent for the research (No. 2650/IV-12).

### 2.3. Research Instrument 

The questionnaire created was based on the questionnaires from similar research on PA during pregnancy [41,42,43]. The questionnaire consisted of 85 questions divided in five sections: social characteristics (age, place of residence, marital status, education, employment status, self-rated health, financial status), anthropometric characteristics (height, weight, waist circumference), pregnancy and lifestyle characteristics (planning a pregnancy, symptoms present during the first trimester, and diet), and (leisure-time PA in three months before pregnancy and PA during the first trimester). 

Sections on social characteristics, anthropometric characteristics, and pregnancy and lifestyle characteristics were adapted from the Pregnancy Risk Assessment Monitoring System Phase Seven questionnaire (PRAMS questionnaire) [42]. PA in the three months before pregnancy was assessed with the International Physical Activity Questionnaire (IPAQ) [43]. PA during the first trimester was assessed with the Pregnancy Physical Activity Questionnaire (PPAQ) [41]. The final questionnaire was modified and translated to Serbian by two independent translators following the standard method of translating (forward and backward translation) [44]. Researchers agreed on the final version of the questionnaire, after which a pre-testing on ten women was done. Pre-testing was done to determine whether the questions were clear, concise, understandable, if their order was adequate, and to determine the time needed to fill in the questionnaire (which was approximately 15 min). Assessment of test–retest reliability was done with 20 pregnant women, with whom the retest was performed two weeks after filling in the initial questionnaire, using kappa coefficients, which were ≥0.90. The test–retest reliability of the Serbian version of IPAQ was published elsewhere [45]. 

Anthropometric characteristics (weight, height, and waist circumference) were taken after the participants agreed to participate in the study. All the measurements were done by the lead researcher. Waist circumference was measured with the participant in the standing position, at the end of expiration, with inelastic tape, at the nearest 0.5 cm, and the tape positioned at the middle point between the top of the iliac crest and the lowest palpable rib. 

### 2.4. Variables 

*Level of Leisure-time PA in three months before pregnancy* was assessed through total energy expenditure in metabolic equivalents (METS) [43] using the IPAQ. For calculations, it is considered that energy expenditure during walking is 3.3 METs every minute, energy expenditure during moderate-intensity PA is 4 METs, and during vigorous-intensity PA 8 METs. Total weekly energy expenditure is accordingly calculated using the formula: 

Total energy expenditure (MET-minutes/week) = Time (in minutes) spent in vigorous physical activity per day × number of days per week with vigorous physical activity × 8 + time (in minutes spent) in moderate Physical activity per day × number of days per week with moderate physical activity × 4 + time (in minutes) spent walking per day × number of days per week walking × 3.3.

Based on total energy expenditure in MET-minutes per week, women were classified into three groups: low (<600 MET-minutes per week), moderate (601–3000 MET-minutes per week), or high (>3000 MET-minutes per week) level of PA [43]. 

*Leisure-time physical activity (LTPA)during pregnancy* was presented in MET-minutes per week and calculated based on the recommended calculations for PPAQ of duration and intensity of each type of PA [41]:

Total energy expenditure (MET-minutes/week) = (duration × 3.2 for walking slowly for fun/exercise+ duration × 4.6 for walking quickly for fun exercise + duration × 6.5 for walking up-hill quickly for fun/exercise + duration × 7.0 for jogging + duration × 3.5 for prenatal exercise class + duration × 6.0 for swimming + duration × 4.5 for dancing) × 60.

Duration of each leisure-time PA was given in six categories: none; less than thirty minutes; more than thirty minutes, but less than one hour; between one and two hours; between two and three hours; and more than three hours. These categories were translated into durations: 0, 0.25, 0.75, 1.5, 2.5, 3, which were then computed into average weekly time in these activities. The intensity was calculated based on the specific MET values assigned to each activity (walking slowly for fun/exercise—3.2, walking quickly for fun exercise—4.6, walking up-hill quickly for fun/exercise—6.5, jogging—7.0, prenatal exercise class—3.5, swimming—6.0, and dancing—4.5) [41]. Women were classified into two groups: a group of sufficient and a group of insufficient PA. Insufficient PA was defined as PA of less than 600 MET-minute/week, based on recommendations by WHO which state that the minimal PA for adults, including pregnant women, is 600 MET-minutes/week [46]. 

*The sedentary time before pregnancy* was assessed with the question ‘How many minutes per day do you on average spend sitting?’ using the IPAQ [43]. All values were then translated into hours per week. *The sedentary time during pregnancy* was assessed with questions from PPAQ regarding sedentary activities (‘How many hours per day do you on average spend watching TV/being on the computer outside of work/working on the computer at work, reading books, driving?’) [41]. In the PPAQ questionnaire duration was given in six categories: none; less than thirty minutes; more than thirty minutes, but less than one hour; between one and two hours; between two and three hours; and more than three hours. These categories were translated into durations: 0, 0.25, 0.75, 1.5, 2.5, 3, which were then computed into average weekly time in these activities. 

Education was assessed as the number of years of education, more than 12 years (college/faculty), or 12 years or less (primary/secondary education). Participants were asked to name their place of residence which was then classified by researchers into urban and rural according to the division made by the statistical office of Serbia [47]. Self-perceived financial status was assessed with the question: ‘How would you describe your financial status?’ (good, average, poor).

### 2.5. Analyzed Variables 

A total of 22 variables were included in the analyses to assess their association with insufficient LTPA. These were: age, nationality, place of residence, years of education, marital status, working status, self-rated financial status, if the pregnancy was planned, parity, nausea in pregnancy, daily fruit intake, daily vegetable intake, chronic disease, height, weight, waist circumference, BMI, PA category based on METS pre-pregnancy, total time before pregnancy spent sedentary, spent walking, spent in moderate or vigorous LTPA before pregnancy. 

### 2.6. Statistical Analyses

Statistical analyses were done with methods of analytical and descriptive statistics. Normality was tested using the Shapiro–Wilk test. Significance of differences between normally distributed continuous variables was examined using the T-test. Chi-square test was used to assess differences in social characteristics, medical, and dietary between the groups of insufficient and sufficient LTPA. All variables which were shown to be significant (*p* < 0.05) were entered into a multivariate logistic regression model with insufficient LTPA as an outcome variable. Analyses were done using Statistical Package for Social Science SPSS for Windows 21.0 (Armonk, NY, USA).

## 3. Results

A total of 175 women were included in the study. Thirteen questionnaires were not properly filled in, and our final analysis included 162 pregnant women. A total of 44/162 (27.2%) women had insufficient LTPA during the first trimester of pregnancy (<600 MET-minutes/week). Average MET-minutes/week during pregnancy in a group of sufficient PA was 1482.88 ± 728.71, while in a group of insufficient LTPA was 372.99 ± 24.09 MET-minutes/week. 

The total of 39.5% of women in a group of insufficient LTPA had a low level of PA before pregnancy, compared to 18.9% of women from the sufficient pregnancy LTPA group, *p* = 0.027.

The groups differed significantly in frequency of more than 12 years of education, self-rated financial status, and total LTPA level before pregnancy (Table 1).

The average number of hours per week spent in walking before pregnancy was significantly higher in the group of sufficient LTPA during the first trimester (4.73 ± 0.44 hours/week vs. 3.17 ± 0.48 hours/week, *p* = 0.006 (Table 2).

Multivariate logistic regression model showed that women with 12 or less years of education had 2.3 (OR: 2.30, 95% CI: 1.05–5.04) times higher odds for having insufficient LTPA during the first trimester. Women who rated their financial status as poor had lower odds for insufficient LTPA (OR: 0.34, 95% CI: 0.14–0.79) compared to women who rated their financial status as good. More hours spent walking before pregnancy were associated with lower odds for insufficient LTPA during pregnancy (OR: 0.87, 95% CI: 0.77–0.99) (Table 3).

## 4. Discussion

Our study showed that almost one-third of women in their first trimester (27.2%) had insufficient LTPA. Insufficient LTPA was associated with lower educational level, higher self-perceived financial status, and time spent walking during the three months before pregnancy. 

More than one-quarter of the pregnant women (27.2%) were insufficiently active during early pregnancy in our study, which is similar to the results from the Omega study in which 25% of women were insufficiently active during early pregnancy [16]. Other studies found a higher prevalence of insufficient PA during pregnancy [32,48]. A study among pregnant women in Singapore found insufficient PA among 34.3% of participants [32], while a national study in the United States showed that the prevalence of insufficient LTPA during early pregnancy is 43% [48]. The higher prevalence of insufficient PA in United States can be associated with an increase in the prevalence of physical inactivity among the adult female population in the U.S. from 19.1% in 1988 to 51.7% in 2010 [49]. However, the data from Serbia differ. The prevalence of physical inactivity among adults in Serbia is 68% [50], just over one-quarter of our sample were insufficiently active. It was suggested that pregnancy might trigger the adoption of the risk-reducing behaviors, like a lifestyle change, same as other life changing events [51], which might explain the difference between the studies. 

One of the most commonly identified factors associated with LTPA level during pregnancy, in all trimesters, including the first [52], was LTPA level before pregnancy. That being said, a prospective study conducted among 1175 healthy pregnant women in Spain showed that almost one-half of pregnant women who were sufficiently active before pregnancy did not stay active during pregnancy [53]. Our results stressed the association between sufficient/insufficient LTPA in pregnancy and only hours spent walking before pregnancy, not spent in moderate or vigorous PA. Our results might suggest that women, who used to walk before pregnancy, can continue this throughout the pregnancy, while those who engage in moderate or vigorous-intensity exercises tend to stop this. This might be due to fear of injuries, but also due to the feeling of decreased maneuverability during pregnancy, when women do not feel safe exercising [37,54].Another reason might be that there is a strong traditional belief that walking in pregnancy can make childbirth easier.

Although we did not examine PA levels during the second trimester, some studies have shown an increase in LTPA during the second trimester, like research done on pregnant women in all three trimesters in Denmark which showed a higher number of footsteps per day during the second trimester compared to both third and first trimester [55]. Others have shown a progressive decline in the prevalence of sufficient LTPA throughout the pregnancy [56,57,58]. Having that in mind, and a possibility that the prevalence of insufficient LTPA might only increase later in pregnancy, the exact identification of walking time as an independent predictor of sufficient LTPA in pregnancy can direct health promotion efforts and physician guidelines for LTPA in pregnancy, and furthermore, improve the prevalence of women who comply with those guidelines. 

A low educational level was previously associated with a decrease of LTPA during pregnancy, both in high and in middle-income countries [53] and our study showed that it is associated not only with LTPA level decrease but with it being insufficient to maintain health. Similar to our results, studies have also associated low pregnancy LTPA with higher income [56,59]. These two findings might only seem conflicting. Pregnant women of poor self-perceived socio-economic status might have to walk more for transportation, rather than use a car or even public transport, and therefore report a higher number of minutes spent walking during pregnancy. Women who have a higher education likely have a higher consciousness about the necessity of a healthy lifestyle during pregnancy.

Parity and pre-pregnancy BMI were also identified as factors associated with the lifestyle of pregnant women [58,59]. Women with children are expected to have less time for LTPA, while women with higher BMI are thought to more often have a sedentary lifestyle, and are expected to continue with a sedentary lifestyle in pregnancy as well [52]. Our study did not find these associations since both pre-pregnancy BMI and parity were not associated with insufficient LTPA during pregnancy. We did not find any association with pregnancy symptoms, such as nausea and reduction of LTPA, although the prevalence of nausea was a bit higher in the insufficient PA group. Our finding differs to the study on 1171 women in Singapore which showed that nausea in the first trimester is associated with the reduction of LTPA during pregnancy [60].

The main limitation of this study is its retrospective design as there could have been a recall bias for leisure-time PA. Another limitation is that the study was conducted in only one center in Serbia, and the sample might not be representative of the entire population. We relied on self-reported data, so there could be some bias, as women might have overestimated their LTPA levels both in pregnancy and before pregnancy. Another limitation is a cross-sectional design that does not allow the establishment of a causal relationship between the variables. Nonetheless, this is one of the few studies that have examined pre-pregnancy and first trimester LTPA at the 12th week of gestation and not during the 24–28th week period or postpartum and these data can be very valuable.

## 5. Conclusions

Results from this study show that almost one third of the pregnant women in Serbia are insufficiently active during the first trimester. Sufficient LTPA was associated with hours spent walking before pregnancy. This allows health care professionals to design their advice for women in their reproductive period or women who are planning pregnancy and direct them to walking as it seems to be the type of LTPA that is usually sustained during pregnancy. Walking is a type of PA that is easily integrated into daily routines and is resistant to the common barriers to PA that many pregnant women face. Due to this resistance to common barriers, walking can provide very important benefits for maternal and child health. It is the ideal type of PA for all women, including those who were insufficiently active or sedentary before pregnancy. Community-based approaches to create communities that foster walking for the entire population may be needed to support the conditions for walking among pregnant women. Our study also showed the association of insufficient LTPA during the first trimester with educational level and self-perceived financial status. Health care professionals can easily identify women with a higher likelihood for insufficient LTPA through questions on social characteristic described in this study and pay more attention to advising these women on a healthy lifestyle during pregnancy. 

## Figures and Tables

**Table 1:** Sufficiently and insufficiently active participants.

	Sufficient LTPA	Insufficient LTPA	*p*-Value
Age (mean ± SD)	31.42 ± 5.29	32.16 ± 4.99	0.426
Nationality *N* (%)			
Serbian	117 (99.2)	44 (100)	
Other	1 (0.8)	0 (0)	0.540
Place of residence *N* (%)			
Urban	110 (94.8)	38 (86.4)	
Rural	6 (5.2)	6 (13.6)	0.07
Marital status *N* (%)			
Married/permanent relationship	118 (100)	44 (100)	
Single	0	0	/
Working status *N* (%)			
Employed	98 (83.1)	39 (88.6)	
Unemployed	20 (16.9)	5 (11.4)	0.381
Years of education *N* (%)			
≤12 years of education	38 (32.2)	23 (52.3)	
>12 years of education	80 (67.8)	21 (47.7)	**0.019**
Self-rated financial status *N* (%)			
Poor	14 (12.2)	7 (16.3)	
Average	66 (57.4)	14 (32.6)	
Good	35 (30.4)	22 (51.2)	**0.019**
Planned pregnancy *N* (%)	85 (72)	37 (84.1)	0.113
Nausea *N* (%)	80 (67.8)	33 (75.0)	0.375
Has children *N* (%)	55 (46.6)	25 (56.8)	0.248
Daily fruit intake *N* (%)	79 (66.9)	31 (70.5)	0.671
Daily vegetables intake *N* (%)	67 (56.8)	22 (50.0)	0.440
Chronic disease *N* (%)	34 (28.8)	13 (29.5)	0.927
Height in cm (mean ± SD)	167.65 ± 6.43	168.14 ± 5.73	0.660
Weight in kg (mean ± SD)	68.53 ± 15.96	66.81 ± 14.35	0.533
Waist circumference in cm (mean ± SD)	78.88 ± 11.90	77.25 ± 12.42	0.569
BMI in kg/m^2^ (mean ± SD)	24.29 ± 4.95	23.64 ± 5.05	0.457
PA level before pregnancy *N* (%)			
Low	21 (18.9)	17 (39.5)	
Moderate	63 (56.8)	17 (39.5)	
High	27 (24.3)	9 (20.0)	**0.027**

LTPA = leisure time physical activity; SD = standard deviation; BMI = body mass index; PA = physical activity; All bold values are statistically significant.

**Table 2:** Physical activity and sedentary time before and during pregnancy among sufficiently and insufficiently active participants.

	Sufficient LTPA (Mean ± SD)	Insufficient LTPA (Mean ± SD)	*p*-Value
Sedentary before pregnancy (Hours/week)	25.88 ± 2.62	22.97 ± 4.06	0.942
Walking before pregnancy (Hours/week)	4.73 ± 0.44	3.17 ± 0.48	**0.006**
Moderate recreational physical activity before pregnancy (Hours/week)	5.37 ± 0.5	4.83 ± 0.74	0.326
Vigorous physical activity before pregnancy (Hours/week)	4.80 ± 0.44	3.57 ± 0.54	0.379

LTPA = leisure time physical activity; All bold values are statistically significant.

**Table 3:** Logistic regression analyses with insufficient leisure-time physical activity during pregnancy as an outcome variable.

Characteristic	OR (95% CI)
Years of education	
≤12 years of education	**2.30 (1.05–5.04)**
>12 years of education	1.0 (reference category)
Self-rated financial status	
Poor	**0.34 (0.14–0.79)**
Average	0.85 (0.27-2.69)
Good	1.0 (reference category)
PA level before pregnancy	
Low	1.01 (0.26-3.91)
Moderate	0.54 (0.18-1.57)
High	1.0 (reference category)
Walking before pregnancy (Hours/week)	**0.87 (0.77–0.99)**

All bold values are statistically significant.
